# ‘Medusa head ataxia’: the expanding spectrum of Purkinje cell antibodies in autoimmune cerebellar ataxia. Part 3: Anti-Yo/CDR2, anti-Nb/AP3B2, PCA-2, anti-Tr/DNER, other antibodies, diagnostic pitfalls, summary and outlook

**DOI:** 10.1186/s12974-015-0358-9

**Published:** 2015-09-17

**Authors:** S. Jarius, B. Wildemann

**Affiliations:** Molecular Neuroimmunology Group, Department of Neurology, University of Heidelberg, Otto Meyerhof Center, Im Neuenheimer Feld 350, D-69120 Heidelberg, Germany

**Keywords:** Autoimmune cerebellar ataxia, Cerebellitis, Paraneoplastic cerebellar degeneration, Autoantibodies, Purkinje cells, Metabotropic glutamate receptor 1 (mGluR1) antibodies, Homer-3 antibodies, Anti-Sj, Inositol 1,4,5-trisphosphate receptor 1 (ITPR1, I3PR) antibodies, Carbonic anhydrase-related protein VIII (CARP VIII) antibodies, Protein kinase gamma (PKCγ) antibodies, Anti-Ca, Rho GTPase activating protein 26 (ARHGAP26, GRAF) antibodies, Glutamate receptor delta2 (GluRδ2) antibodies, Anti-Yo, Cerebellar degeneration-related protein 2 (CDR2) antibodies, Cerebellar degeneration-related protein 2-like (CDR2L) antibodies, Purkinje cell antibody 2 (PCA-2), Anti-Tr, Delta notch-like epidermal growth factor-related receptor (DNER) antibodies, Anti-Nb, Anti-AP3B2, Neuronal adaptin-like protein (beta-NAP) antibodies, Voltage-gated calcium channel (VGCC) antibodies

## Abstract

Serological testing for anti-neural autoantibodies is important in patients presenting with idiopathic cerebellar ataxia, since these autoantibodies may indicate cancer, determine treatment and predict prognosis. While some of them target nuclear antigens present in all or most CNS neurons (e.g. anti-Hu, anti-Ri), others more specifically target antigens present in the cytoplasm or plasma membrane of Purkinje cells (PC). In this series of articles, we provide a detailed review of the clinical and paraclinical features, oncological, therapeutic and prognostic implications, pathogenetic relevance, and differential laboratory diagnosis of the 12 most common PC autoantibodies (often referred to as ‘Medusa head antibodies’ due to their characteristic somatodendritic binding pattern when tested by immunohistochemistry). To assist immunologists and neurologists in diagnosing these disorders, typical high-resolution immunohistochemical images of all 12 reactivities are presented, diagnostic pitfalls discussed and all currently available assays reviewed. Of note, most of these antibodies target antigens involved in the mGluR1/calcium pathway essential for PC function and survival. Many of the antigens also play a role in spinocerebellar ataxia. Part 1 focuses on anti-metabotropic glutamate receptor 1-, anti-Homer protein homolog 3-, anti-Sj/inositol 1,4,5-trisphosphate receptor- and anti-carbonic anhydrase-related protein VIII-associated autoimmune cerebellar ataxia (ACA); part 2 covers anti-protein kinase C gamma-, anti-glutamate receptor delta-2-, anti-Ca/RhoGTPase-activating protein 26- and anti-voltage-gated calcium channel-associated ACA; and part 3 reviews the current knowledge on anti-Tr/delta notch-like epidermal growth factor-related receptor-, anti-Nb/AP3B2-, anti-Yo/cerebellar degeneration-related protein 2- and Purkinje cell antibody 2-associated ACA, discusses differential diagnostic aspects and provides a summary and outlook.

## Introduction

Autoimmune cerebellar ataxia (ACA) represents an important differential diagnosis in patients presenting with signs and symptoms of cerebellar disease. Alongside multiple sclerosis and acute disseminated encephalomyelitis, autoantibody-associated disorders of the central nervous system (CNS) are the most common cause of ACA. While ACA is a rare manifestation in some antibody-related disorders, such as in aquaporin-4 (AQP4) antibody-associated neuromyelitis optica (NMO), it is the most frequent or exclusive presentation in others. To date, around 30 different autoantibodies targeting brain antigens have been reported in patients with ACA, many of which are of paraneoplastic origin.

When tested by immunohistochemistry (IHC) using cerebellum tissue sections, 12 of those antibodies show a staining pattern resembling a Gorgon head caused by binding of IgG to Purkinje cell (PC) somata and dendrites and are therefore often referred to as ‘Medusa head’ antibodies. Most of these antibodies are involved in regulating calcium homoeostasis in PCs.

In part 1 of this series of articles, we focused on anti-metabotropic glutamate receptor 1 (mGluR1)-, anti-Homer protein homolog 3 (Homer-3)-, anti-Sj/inositol 1,4,5-trisphosphate receptor (ITPR1)- and anti-carbonic anhydrase-related protein VIII (CARP VIII)-associated ACA [1]. The second part covered anti-protein kinase C gamma (PKCγ)-, anti-glutamate receptor delta-2 (GluRδ2)-, anti-Ca/RhoGTPase-activating protein 26 (ARHGAP26)- and anti-voltage-gated calcium channel (VGCC)-associated ACA [2]. In the present, third part of our article series, we focus on anti-Tr/delta notch-like epidermal growth factor-related receptor (DNER)-, anti-Nb/AP3B2-, anti-Yo/cerebellar degeneration-related antigen 2 (CDR2)-, anti-cerebellar degeneration 2-like (CDR2L)- and Purkinje cell antibody 2 (PCA-2)-positive ACA, discuss diagnostic pitfalls and present a summary and outlook.

## Anti-Yo/CDR2/CDR62 (PCA-1)

### Clinical, paraclinical and epidemiological features

Anti-Yo antibodies were first reported by Greenlee and Brashear in two patients with paraneoplastic cerebellar degeneration (PCD) in 1983 [3]. Since then, hundreds of cases have been identified, rendering anti-Yo one of the most common paraneoplastic antibodies. Clinically, most patients present with a subacute and mostly severe pancerebellar syndrome with truncal and appendicular ataxia, dysarthria and (mostly downbeat) nystagmus; other signs and symptoms may be present, with mild long-tract involvement, peripheral neuropathy, dysphagia, diplopia, vertigo and cognitive impairment being the most common ones [4]. Median age at onset is 61 years (range, 26–85) [4]. Almost all patients are female, though a few male patients have been described [5–9].

MRI often shows cerebellar atrophy after some weeks or months but may be normal at disease onset (17/55 cases in reference [4]). CSF often shows lymphocytic pleocytosis, oligoclonal bands, an increased CSF/serum ratio and elevated total protein levels [4, 10].

#### Tumour association

Anti-Yo antibodies are usually associated with malignant gynaecological tumours (ovary, breast, mesovarium, fallopian tube, uterus or cervix), which are often confined to the involved organs or local lymph nodes [4]. Rarely, other tumours have been found, including lung cancer [4]. In the few male anti-Yo-positive patients, PCD was associated with adenocarcinomas (prostate, esophagus, stomach or intestine) [5–9].

In the majority of patients, the onset of PCD precedes the tumour diagnosis by months or even years [4]; accordingly, regular screening for malignant tumours is required in confirmed, positive cases without tumour at first presentation [11]. Sometimes, carcinomas remain occult and are detected only upon autopsy, possibly due to an effective anti-Yo-related anti-tumour immune response and, though rarely, no tumour is found [4]. In the latter case, preventive hysterectomy and salpingo-ovarectomy have been proposed [12] if symptoms are severe and do not respond to treatment and provided the positive test result has been confirmed in a second assay.

Anti-Yo antibodies were absent in patients with ovarian or breast cancer but no neurological symptoms in one study (*n* = 79) [4] but were found in a small subset of patients (~2 %) in another one [13]. Anti-Yo was not detected in 150 control subjects who had neither PCD nor cancer [4, 14].

#### Outcome and prognosis

Given that the disease usually takes a subacute, rapidly progressive course, which often results in complete loss of PCs and, accordingly, substantial disability within weeks or a few months, early treatment is crucial. Aggressive tumour therapy is the mainstay of disease management. Immunosuppression has mostly no or only minor effect [4, 15, 16], although PEX, IVIG, rituximab, steroids and cyclophosphamide were reportedly beneficial in some cases [17–20], especially if treatment was initiated shortly after disease onset.

Peterson et al. [4] found that 37 out of 48 assessable patients were unable to walk or sit unassisted at final follow-up. The median survival time in anti-Yo-positive patients is 22 months; however, long-term survival (up to 164 months) has been reported, too [15]. Prognosis may also be determined by the type of the underlying tumour, with breast cancer patients having a better prognosis [15]. PCD rather than tumour progression is the cause of death in around 40 % [15].

#### Antigen

**Fig. 1:**
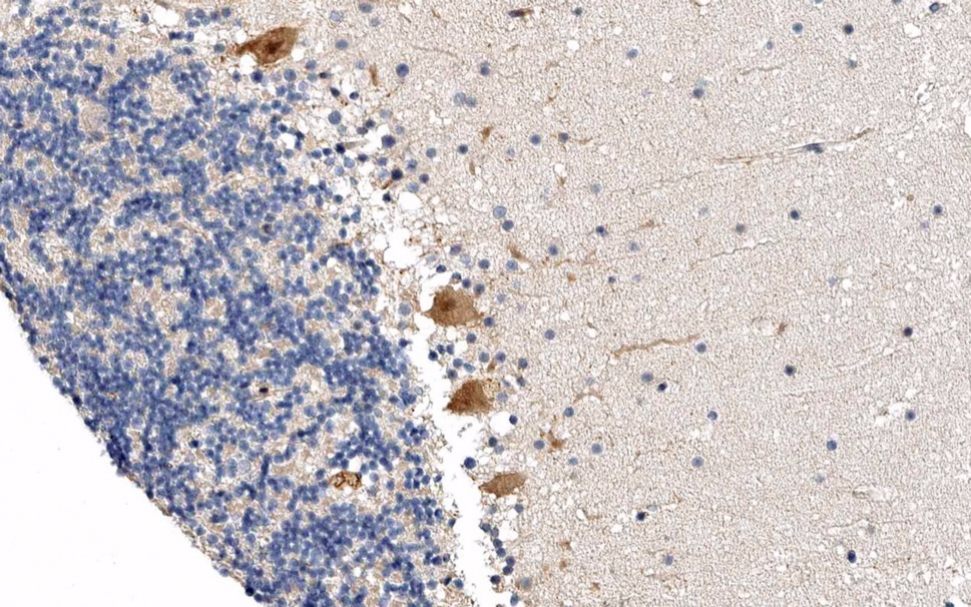
Expression of CDR2 in the human cerebellum as demonstrated by IHC (modified image from the *Human Protein Atlas* image database [33])

Anti-Yo-positive sera recognize CDR2 (cerebellar degeneration-related 2; less commonly termed CDR62), a 62-kDa protein with a helix leucine zipper (HLZ) and a zinc finger motif [21, 22] that is widely expressed in gynaecological tumours [23] but mainly restricted to cerebellum (Fig. 1), brainstem. In healthy tissue it is mainly restricted to cerebellum, brainstem and testis [24–26] and testes in healthy tissue [24–26]. The antibodies partly target the leucine zipper motif of CDR2, giving rise to the possibility of cross-reactivity with proteins containing such a motif [27]. At lower magnitude and inconstistently, anti-Yo-positive sera were shown to recognize a second, 34-kDa onconeuronal protein in cerebellum or PC Western blots which contains a unique six amino acid (aa) consensus sequence (L/FLEDVE) [28–30].

Moreover, anti-Yo sera have been reported to bind to CDR3 [31], a protein similar to CDR2, and, in 85 %, to CDR2L (cerebellar degeneration-related 2-like) [14, 32]. CDR2L is expressed in PCs at a higher level than CDR2 [14] and has been detected in a variety of human tumours [14, 33], including ovarian and breast cancer cells.

#### Immunohistochemistry

**Fig. 2:**
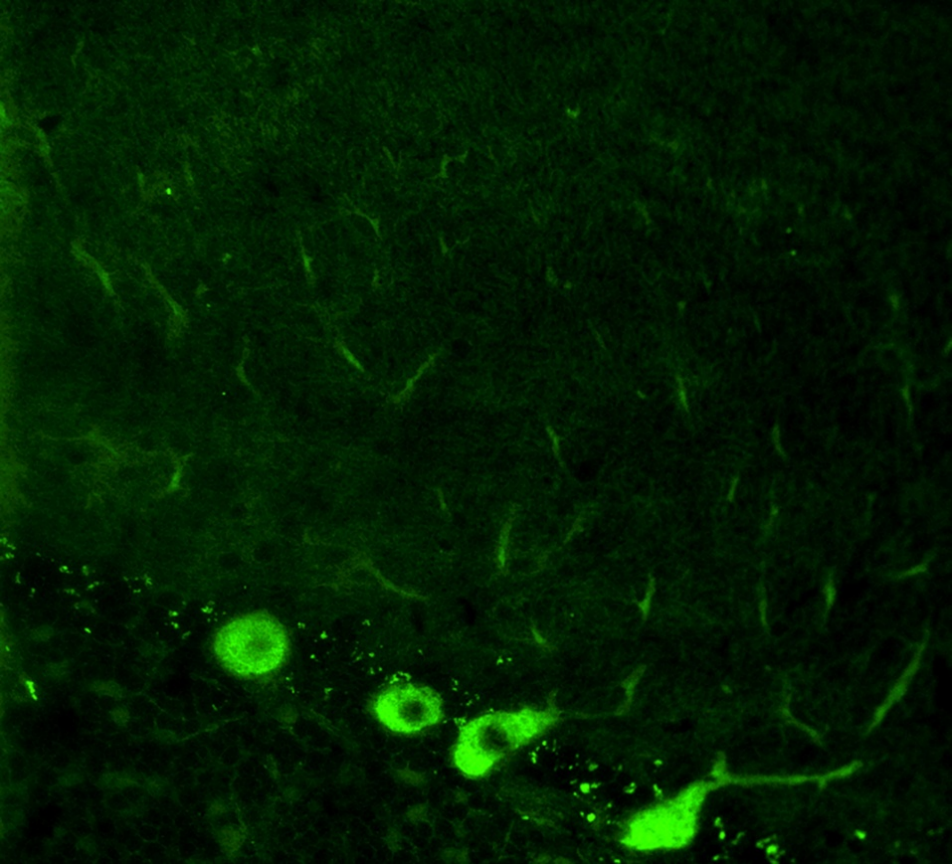
Binding of anti-Yo (PCA-1) from a patient with ACA to a mouse cerebellum tissue section. The patient antibody was detected by use of a goat anti-human IgG secondary antibody labelled with Alexa Fluor@488 (*green*)

The antibody stains the cytoplasm of the PC somata in a typical coarse, granular pattern and spares the nucleus. Depending on titres, tissue donor species and fixation and staining methods, additional binding to the PC dendritic arbour and, weakly, the peripheral PC dendritic branches can be observed (Fig. 2) [3, 34–36]. However, no axonal staining is observed. In addition, neurons in the deep cerebellar nuclei are strongly stained by anti-Yo in human tissue [34, 36].

According to our experience and that of other groups [31], PCs are the main antibody target. However, additional immunostaining of human and rat basket and stellate neurons by anti-Yo-positive sera has been reported [36].

Outside the cerebellum, binding to (large, cytoplasm-rich) neurons in the brain, brainstem, retina, anterior horns, sensory and sympathetic ganglia, and the myenteric plexus has been observed as well as staining of the cytoplasm of Schwann cells [34, 37].

At the subcellular level, anti-Yo was found to bind to clusters of ribosomes, orderly arrays of stacked parallel cisternae of the granular endoplasmic reticulum and the trans-face of the vesicles of the Golgi complex in the perikaryon and dendritic processes of PCs [38]. A second study found reactivity to both the rough and smooth endoplasmic reticulum and polyribosomes in human and rat cerebellar Purkinje cells, other neuron cell bodies and Schwann cells [39], while a third study reported immunostaining of free and membrane-bound ribosomes, but not of the endoplasmic reticulum lumen, smooth endoplasmic reticulum, Golgi complex, mitochondria or nucleus [40]. Whether these minor discrepancies were caused by methodological differences among studies or due to different antigen or epitope specificities between the respective patients is unclear. Outside the CNS, staining of the adrenal medulla and of epithelial cells of the renal glomerulus has been observed [34].

#### Antigen-specific assays

Numerous commercial and in-house assays are available, including line or dot immunoblot (IB) assays, ELISAs, immunoprecipitation (IP) assays and cell-based assays (CBAs). Most immunoassays use exclusively the major antigen CDR62 as test substrate since the minor 34-kDa reactivity is associated with CDR62 in all cases. Systematic studies comparing the different assays are currently lacking but are warranted. Storstein et al. [41] reported higher sensitivity of an anti-CDR2-specific IP assay than of an IB and a fluorescence-based IHC assay, but also a lower specificity. The same group also found a higher sensitivity of IP for detecting anti-CDR2L [14]. CDR2- and a CDR2L-specific CBAs (Euroimmun, Luebeck, Germany) are available at the authors’ institution for use in scientific studies.

#### CSF testing

Stich et al. found evidence for intrathecal IgG synthesis, as indicated by an elevated antibody index in 5/5 patients with anti-Yo-associated PCD [10, 42]. In a previous study, 9/9 patients with PCD and anti-Yo serum antibodies were found to have CSF-restricted CDR2-specific oligoclonal bands [10].

#### Association with other autoantibodies

Anti-Yo are not typically associated with other well-established paraneoplastic anti-CNS antibodies. However, a single study reported antibodies to 20S proteasome proteins in 8/14 patients with anti-Yo-positive PCD [43], and a second one found antibodies to an uncharacterized protein called coiled-coil domain-containing protein 104 (CCDC104) in around 10 % of anti-Yo-positive patients [44]. However, the clinical relevance of these co-reactivities is currently unknown.

#### Pathogenetic relevance

Pathologically, anti-Yo-positive PCD is characterized by a massive loss in PCs, which can be accompanied by reactive Bergman cell gliosis [45, 46]. In some, but not all cases—possibly depending on the time between onset and autopsy or biopsy [47]–, immune cell infiltrates are present [45, 46]. Cell types reported included both T and B cells, plasma cells and macrophages/microglial cells [31, 46]. In accordance with the fact that CDR2 is also expressed outside the cerebellum, inflammatory infiltrates have been observed in the medulla, pons and/or cerebrum (partly symptomatic [48]) as well as axonal loss in the spinal cord [45, 47]. In the few cases examined histopathologically, no anti-Yo antibodies could be detected at the lesion site. Given the rapid loss of PCs, it is possible that these examples of ‘non-inflammatory PC degeneration’ represent final burn-out stages [4]. This would also explain the lack of immunoglobulin and complement deposits found in some studies [45, 46].

The currently most widely accepted pathogenetic scenario is that the ectopic expression of CDR2 in tumour tissue results in loss of immunotolerance against that protein, resulting in secondary damage of neurons naturally expressing CDR2, which is mediated by onconeuronal antibodies and sensitized T cells [31, 49].

The exact mechanism by which anti-Yo could cause PC damage is unclear. Anti-Yo mainly belong to the complement-activating subclass 1 (rarely, IgG2, IgG3, IgM or IgA anti-Yo antibodies are present) [50–53] and are produced intrathecally [10, 42]. However, CDR2 is believed to be a mainly cytoplasmic protein not present in the membrane and thus not accessible to circulating IgG [38, 40]. By contrast, CDR2L, which has recently been proposed to be an additional target antigen of anti-Yo, is, at least in transfected HeLa cells, membrane bound according to one study [14]. This would be of particular interest given that Eichler et al. [14] found PCD only in anti-Yo patients who reacted both to CDR2 and CDR2L. However, it is not known to date whether CDR2L is membrane linked in PCs in vivo as well. Of note, Rodriguez et al. [38] found binding of anti-Yo-like antibodies from patients with gynaecological tumours to patches of external plasma membrane by electron microscopy and discussed whether the antigen might transiently reach the plasma membrane via the Golgi complex or by being incorporated in synaptosomes. However, at the time of testing, the target antigen of anti-Yo was unknown and no Western blot data was provided [38].

Alternatively, intracellular uptake of anti-Yo by PCs could cause pathogenic effects. Viable PCs have been demonstrated to internalize and accumulate serum, CSF or purified anti-Yo in slice (organotypic) culture experiments as well as in live animals after blood barrier disruption or direct injection into the brain [54–58]. By contrast, control IgG was reported to be rapidly cleared from PCs [56, 57]. Incorporation of myeloma light chains by PCs suggests that human neurons might be able to incorporate IgG as well [59]. Interestingly, in one study, the uptake of IgG could be blocked by colchicine [14].

While Greenlee et al. [57] found that intracellular accumulation of anti-Yo, but not of normal IgG, was followed by PC death after 72–144 h without comprising other cells, no cell death was observed in two previous studies [54, 55]. However, differences in observation times after IgG injection and detection methods might account for that discrepancy [57].

Intracellular anti-IgG could theoretically disturb CDR2- or CDR2L-dependent pathways. CDR2 contains leucine zipper and zinc finger motifs as commonly found in transcription factors and could thus influence protein expression [22]. Of note, CDR2 has been shown to co-localize and co-precipitate with c-Myc, which regulates the expression of more than 10 % of all genes by binding to enhancer box sequences and by recruiting histone acetyltransferases and has, thus, been implicated in important cell functions, including cell cycle progression and apoptosis. In vitro, anti-Yo sera were shown to block the interaction of the two proteins, preventing CDR2-mediated down-regulation of c-Myc-dependent transcription and of sequestration of c-Myc in the neuronal cytoplasm, a mechanism that could lead to PC death by apoptosis [60, 61]. In addition, CDR2 has been implicated in inhibiting NFBκB-dependent transcription in neurons [62] and proposed to regulate the nuclear helix-loop-helix leucine zipper protein MRGX, which has been implicated in cell growth, DNA repair, cell ageing and apoptosis. Overexpression of MRGX in T98G glioblastoma cells led to morphological changes and cell death, which could be prevented by CDR2 [27]. On the other hand, two recent studies did not find signs of apoptosis following anti-Yo uptake by PCs using TUNEL (TdT-mediated dUTP-biotin nick end labelling) and active cleaved caspase-3 staining [32, 57].

An immune electron microscopy study suggested localization of the anti-Yo antigen on the ribosome and thus regulation of protein synthesis rather than gene expression in the nucleus [40].

A recent study investigated the pathological role of CDR antibodies on calcium homeostasis in PCs using cerebellar organotypic slice cultures [32]. In accordance with previous studies, the authors found that PCs rapidly incorporated anti-Yo. Interestingly, this was associated with up-regulation of calcium-dependent PKCγ and the voltage-gated calcium channel Cav2.1, two other autoantigens in PCD, and of the calcium-dependent protease calpain-2 (calpains have been involved in LTD and apoptosis). It was also associated with down-regulation of the cytoplasmic calcium-buffering protein calbindin D28K and of the P/Q-type (VGCC)-modulating PC-specific protein 2 (L7) [32]. Moreover, CDR2 and calbindin were found to co-immunoprecipitate [32]. Morphologically, anti-Yo uptake was associated with reduced dendritic PC arborizations [32]. Anti-Yo could thus exert pathogenic effects by dysregulating cell calcium homeostasis, which would provide a rationale for neuroprotective therapies.

However, passive transfer experiments with anti-Yo injected intraventricularly or into the brain parenchyma have not been successful thus far [54, 58, 63]. Similarly, active immunization experiments with recombinant Yo fusion protein [64] or cDNA [65] resulted in the production of anti-CDR2 antibodies but neuronal damage was not observed.

This has led to the notion that PCD is mainly caused by T lymphocytes. PCs do express MHC class I and could thus present CDR2 peptides [66]. Indeed, Albert et al. [67] detected expanded populations of major histocompatibility complex class I-restricted CDR2-specific, cytotoxic T lymphocytes (CTLs) in the blood of three PCD patients using primary human cells in a recall assay. A potent CTL response could also be elicited by cross-presentation of apoptotic HeLa cells used as a source of CDR2 by dendritic cells. The presence of CDR2-reactive CTLs in patients with PCD was confirmed in several [49, 68, 69], yet not all [70], studies. Of note, T cells transfected with the respective, CDR2-specific T-cell receptors were shown to be capable of destroying tumour cells [69]. A role of regulatory T-cell dysfunction has been proposed [71].

On the other hand, CDR2-specific CTLs were not found in all studies [70, 72] and CDR2-specific T cells induced by active immunization with cDNA as well as transfer of lymphocytes from patients with anti-Yo-associated PCD did not cause PC damage in two studies [58, 65].

#### Molecular genetics

To date, no mutations in the CDR2 and CDR2L genes have been described in patients with SCA.

## Anti-Nb/AP3B2 (beta-NAP)

### Clinical, paraclinical and epidemiological features

In 1989, Darnell and colleagues reported on a PC antibody in a 35-year-old woman with a subacute pancerebellar syndrome developing over 1 week and no tumour at the time of testing [73, 74]. The patient presented with severe and incapacitating truncal and appendicular ataxia, titubation of the head and trunk at rest and nystagmus. The disease rendered her bedridden within 2 months. According to the authors, the cerebellar syndrome could not be distinguished from that seen with anti-Hu- or anti-Yo-positive PCD.

Extracerebellar symptoms included diffuse hyperreflexia with bilateral extensor plantar responses. Formal neuropsychiatric testing did not reveal any cognitive impairment. Neither CSF analysis (3 white cells/μl; normal protein; no OCBs) nor brain and spinal cord MRI revealed pathological findings.

#### Tumour association

In the only patient reported thus far, no tumour was detected at last follow-up [74]; however, the patient had a history of hysterectomy and salpingo-oophorectomy [31].

#### Outcome and prognosis

The patient was still alive 3 years after disease onset, i.e. at last follow-up, but was unable to stand or sit and to feed or wash herself. Apparently, no immunotherapy had been tried [74].

#### Antigen

In Western blots employing human PC and cerebellar cortex protein extracts, respectively, the patient’s serum IgG was found to recognize three bands of approximately 150, 120 and 65 kDa. Of note, binding of IgG to a band migrating at precisely the same molecular weight as the 150-kDa PC protein was detected in a Western blot analysis of tumour cell lines (SCLC, melanoma, neuroblastoma), leaving the possibility of a paraneoplastic syndrome caused by an occult neuoectodermal tumour.

**Fig. 3:**
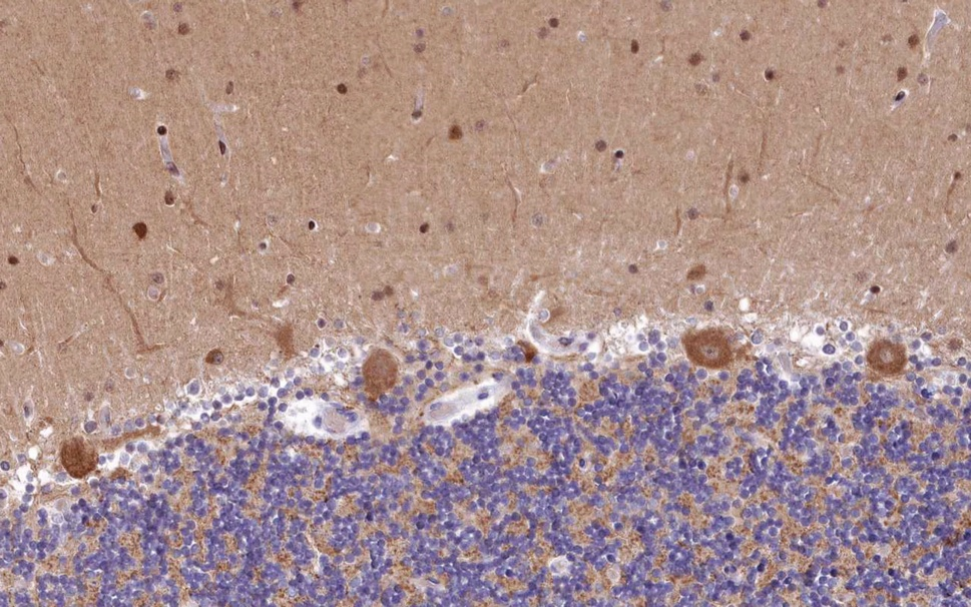
Expression of AP3B2 in the human cerebellum as demonstrated by IHC (modified image from the *Human Protein Atlas* image database [33])

The target antigen was later identified as a neuron-specific vesicle coat protein, named neuronal adaptin-like protein (beta-NAP) [75] and now termed AP3B2 (see Fig. 3 for expression of AP3B2 in the human cerebellum). Beta-NAP forms part of the adaptor complex 3 (AP3). AP3 is a heterotetramer consisting of two large adaptins (AP3D1 and AP3B2, or AP3B1), a medium adaptin (AP3M1 or AP3M2) and a small adaptin (APS1 or AP3S2). Two variants of AP3 exist, the ubiquitously expressed AP3A and the neuron-specific AP3B. AP3B has been implicated in the normal formation and function of some synaptic vesicles. Only the neuronal form is capable of forming synaptic vesicles from endosomes [76]. Mice lacking the μ3B subunit of AP3B morphologically show synaptic abnormalities and functionally impaired GABA release due to a reduction in vesicular GABA transporter [77]. They also present with compromised synaptic zinc stores and synaptic vesicle targeting of membrane proteins involved in zinc uptake (ZnT3 and ClC-3) [78] These findings suggest that AP3B is involved in controlling the levels of selected membrane proteins in synaptic vesicles. Grabner et al. [79] found a correlation between AP3B expression levels in neurons and the neurotransmitter content of individual vesicles as well as with dense-core vesicle size, providing additional evidence for a role of AP3 in neurotransmitter release; the authors located AP3 to the trans-Golgi network and immature secretory vesicles and proposed that the protein is involved in the formation of neurosecretory vesicles.

Of note, beta-NAP transmigrates to the membrane during calcium influx in neurons, linking its subcellular expression profile to intracellular calcium levels and thus to the other antigens discussed in this section [75].

#### Immunohistochemistry

As with anti-Hu, anti-Ri, and anti-Ca, the immunofluorescence pattern was named after the index patients’ initials or code (Nb). In an avidin-biotin peroxidase IHC assay employing frozen sections of human cerebellum, anti-Nb bound predominantly to the PC somata but also to the molecular layer [74]. The antibody reacted with the cytoplasm and nucleoli of PCs, while anti-Hu spared the nucleoli. In contrast to the coarse staining usually seen with anti-Yo, anti-Nb diffusely stained the PC cytoplasm [74, 75]. In addition, anti-Nb caused low-level diffuse staining of the cortex and intense staining of very rare cells in the granular and molecular layer in that study. In the cerebral cortex, the antibody predominantly reacted with neurons in layer VI with only little fluorescence in layer I, while no such gradient was found with anti-Hu.

A follow-up study by Newman et al. [75] also found reactivity of PC somata and processes. In addition to PCs and cortical neurons, the authors reported high-level expression of beta-NAP in hippocampal neurons, too [75]. No expression in glial cells or in cells outside the CNS was observed.

#### Antigen-specific assays

No beta-NAP-specific immunoassays have been published thus far.

#### CSF testing

Anti-Nb were present in the index case at a titre of 1:5000 in the serum and at a titre of 1:100 in the CSF. Similar results were obtained in WB assays of human PCs and human cerebral cortex, respectively (serum 1:1000; CSF 1:100). Given the reportedly normal CSF analysis (including normal protein levels), the relatively high CSF titres as compared to serum are indicative of possible intrathecal anti-Nb synthesis (negative total-IgG OCBs do not exclude intrathecal synthesis of specific autoantibodies [42, 80–84]).

#### Association with other autoantibodies

In the index patient, anti-Nb was associated with an increase in circulating immune complexes, but no further co-existing autoantibodies were reported [74].

#### Pathogenetic relevance

The pathogenetic relevance of anti-Nb has not been investigated yet. Darnell et al. [74] described a cytoplasmic or membrane location of the target antigen. Newman et al. indeed proposed temporary membrane recruitment of beta-NAP from cytoplasmic pools in the nerve terminals and cell body via endosome-derived vesicles upon neuronal depolarization-related calcium influx [75]. Whether this would render beta-NAP accessible to extracellular antibodies seems unlikely, however, since the predicted protein sequence does not contain a transmembrane domain.

#### Molecular genetics

While mutations in the beta-NAP gene have not been reported in patients with cerebellar ataxia so far, ataxia-telangiectasia is caused by mutations in the beta-NAP-interacting ATM protein [85]. Given that the index patient presented with hyperreflexia and a positive Babinski reflex, it is of note that mutations in beta-NAP have been proposed to play a role in spastic paraplegia-6 [86].

## Antibodies to unknown antigens or to antigens not related to the glutamate/calcium pathway

Two autoantibodies have been described in patients with ACA that recognize PC somata and dendrites but are not known to affect the glutamate/calcium pathway: PCA-2 [35], the target antigen of which is unknown, and anti-Tr, which has recently been shown to target DNER [87].

## PCA-2

### Clinical, paraclinical and epidemiological features

So far, nine patients with PCA-2 and neurological symptoms have been reported (7 women, 2 men; age: 44–73 years) [35]. Of note, only three of those patients had predominant cerebellar ataxia (2 women, 1 man; age 44, 66 and 73). Symptoms in those patients included wide-based gait, limb ataxia, dysarthria, loss of fine motor control and intention tremor; in one patient, who was also positive for CV2/CRMP5 and neuronal acetylcholine receptor (AChR) antibodies, ataxia was accompanied by dysphagia and constipation [35].

The remaining six patients presented with predominant limbic or brainstem encephalitis, autonomic neuropathy, motor neuropathy, Lambert Eaton myasthenic syndrome and/or syndrome of inappropriate antidiuretic hormone secretion [35]; however, moderate limb and truncal ataxia were additionally present in one of them. Given that PCA-2 was frequently associated with other antibodies, such as anti-Hu, anti-CV2/CRMP5, anti-neuron type AChR, anti-VGCC of the P/Q type and anti-VGCC of the N type, it is conceivable that some of those syndromes were not or not exclusively caused by PCA-2-related autoimmunity.

In addition, PCA-2 were found in 1/58 patients with newly diagnosed SCLC but no neurological symptoms at the time of blood sampling [35].

CSF findings were reported for five patients and included mild lymphocytic pleocytosis (*n* = 3; 18 and 9/μl, 1× non-specified) nd elevated total protein (*n* = 3, 51–59 mg/dl); no data on the frequency of positive OCBs are available [35].

Brain MRI was normal in two at first presentation, one of whom later developed an abnormality in the left medial temporal lobe consistent with encephalitis, and was not reported in the other cases [35].

#### Tumour association

With the exception of a single patient with unknown tumour status, PCA-2 was associated with SCLC in all cases reported so far [35]. Of note, in one of the three patients with PCA-2-positive PCD, the diagnosis of (limited) SCLC could only be made from a lymph node biopsy 1 year after onset.

#### Outcome and prognosis

In one patient with PCA-2 and predominant cerebellar disease, a first course of VP-16 and caroplatin chemotherapy was followed by marked improvement of ataxia. In another patient improvement after therapy with oral cyclophosphamide (100 mg/day) was noted. In the third patient, the outcome was unknown. In the patient with predominant limbic encephalitis and concomitant cerebellar ataxia, seizures ceased under treatment with intravenous and oral steroids, but cognition and balance continued to decline [35].

#### Antigen

The antigen targeted by PCA-2 is still unknown.

#### Immunohistochemistry

**Fig. 4:**
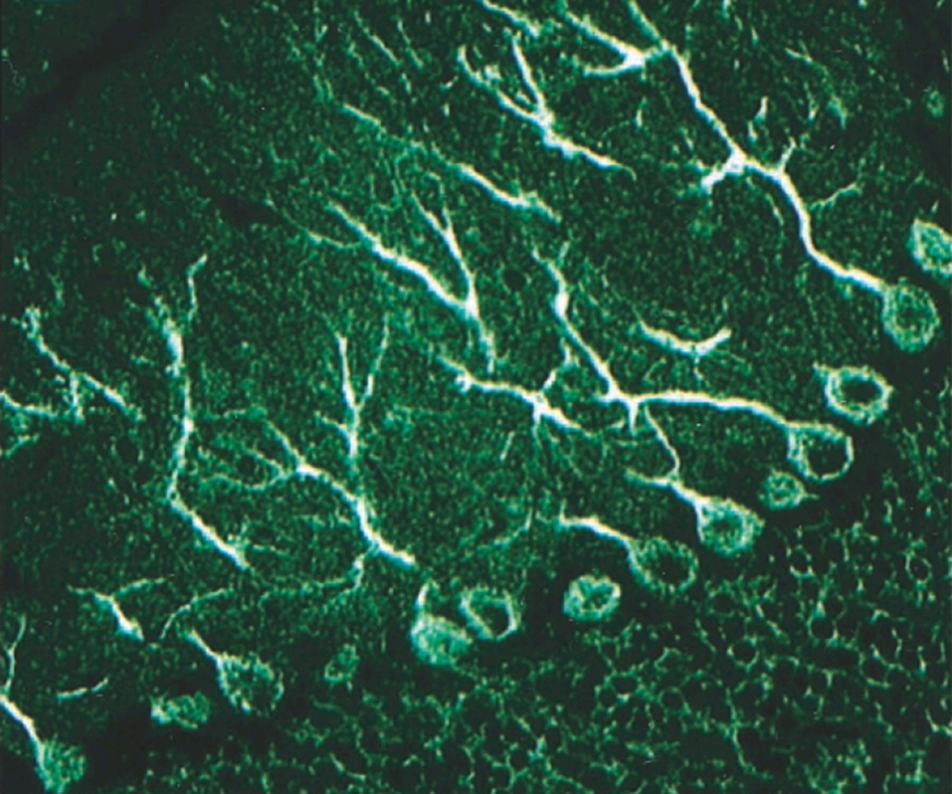
Binding of PCA-2-IgG from a patient with ACA to a formalin-fixed mouse cerebellum tissue section. Note the additional staining of the cytoplasm of cerebellar granular cells (so-called ‘chicken-wire pattern’). A FITC-labelled anti-human IgG secondary antibody was used to visualize bound patient IgG. Image modified from Vernino S and Lennon VA. New Purkinje cell antibody (PCA-2): marker of lung cancer-related neurological autoimmunity. Ann Neurol 2000, 47:297-305; © 2000 by the American Neurological Association; reprinted with kind permission from the publisher

PCA-2 was reported to bind to mouse and human PC dendrites and somata (sparing the PC nucleus) (Fig. 4). In the original publication, it was not specified whether PC axons were targeted as well. The cytoplasmic fluorescence was described as ‘reticular’ as opposed to the coarse PC soma staining observed with anti-Yo (PCA-1) and the punctuate staining seen with anti-Tr/DNER [35]. In addition, prominent staining of the cytoplasm (but not nucleus) of dendate nucleus neurons was observed [35]. Finally, the cerebellar granular layer is stained by PCA-2, yielding a faint ‘chicken-wire pattern’ not usually seen with the other Medusa head antibodies discussed in this review [35].

PCA-2 reportedly also stains neural elements in the mouse gut smooth muscle and neuronal elements adjacent to and innervating arterioles in mouse kidney sections [35].

Whether anti-VGCC contributed to the PC staining in those four patients with concomitant anti-VGCC was not examined [35].

#### Antigen-specific immunoassays

As the antigen is unknown, no antigen-specific assays exist. However, Western blotting of SDS-denatured rat cerebellum protein extracts consistently revealed binding of patient IgG to an unidentified 280-kDa protein in all ten patients analysed [35].

#### CSF testing

CSF was analysed for PCA-2 in two patients, one of whom had PCD. In both cases, relatively high CSF titres as compared to serum were found, suggesting possible intrathecal synthesis.

#### Association with other autoantibodies

In six out of the ten PCA-2-positive patients reported so far, co-existing antibodies to P/Q-type VGCC (associated in LEMS in 2/3), N-type VGCC, neuronal (ganglionic) AChR and/or muscle AChR were present. Two out of the six anti-VGCC-positive patients had cerebellar ataxia, one of whom was in addition positive for anti-CV2/CRMP5 [35].

#### Pathogenetic relevance

So far, no data on the pathogenic impact of PCA-2 exist.

#### Molecular genetics

The antigen of PCA-2 is currently unknown; accordingly, no molecular genetic data exist.

## Anti-Tr/DNER

### Clinical, paraclinical and epidemiological features

The antibody was first described in 1976 by Trotter and colleagues in a 21-year-old woman with subacute PCD and Hodgkin’s disease (HD) [88]. The reactivity was therefore later named anti-Tr (for Trotter) [36]. Graus et al. [36] reported five patients (female:male = 1:4, median age, 36 years [range 14–72]) with anti-Tr, HD and a subacute pancerebellar syndrome with mild to moderate limb ataxia and moderate to severe truncal ataxia and (mostly downbeat) nystagmus in all cases. Of note, extracerebellar symptoms were present in two patients (1× extensor plantar response, 1× retrobulbar optic neuropathy). In two cases, onset of disease was preceded by febrile infection (1× flu-like, 1× tonsillitis). CSF showed pleocytosis in all five cases (median 20 cells/μl [range 12–102]). Brain MRI was normal at initial evaluation but detected cerebellar atrophy in all cases at follow-up.

Bernal et al. [50] described 28 patients (female:male = 1:3.6; median age, 61 years [range 14–75]) with anti-Tr, some of whom had already been included in the previous study by Graus et al. [36]. While 25/28 had an isolated cerebellar syndrome, two additionally had encephalopathy and sensory neuropathy, and one exclusively had symptoms (but no MRI changes) compatible with limbic encephalitis. In 22 cases, the cerebellar syndrome took a subacute and severe course. LP revealed mild CSF pleocytosis in 13/22 patients for whom data were available (median 50 cells/μl [range 14–150]). While the disease took a subacute course in most cases, one patient had only mild, chronic cerebellar ataxia.

A study by Probst et al. [89] tested 38 anti-Tr-positive patients, many of whom may have been already included in previous studies. Of those, 35 had PCD and HD; the remaining samples included 1 patient with PCD without a confirmed tumour, 1 with limbic encephalitis and HD, 1 with paraneoplastic encephalomyelitis and HD, 1 with no neurological symptoms and 1 with missing data.

In a study by De Graaf et al. [87], all 11 patients for whom data were available (7 male, 4 female, median age, 50; range, 12–75) had subacute and severe truncal and limb ataxia with nystagmus and/or cerebellar dysarthria. Brain MRI showed cerebellar atrophy only in 1/11 patients; another patient had white matter lesions.

#### Tumour association

Anti-Tr/DNER antibodies are almost [90] exclusively found in patients with HD. Only a few patients have been reported in whom no HD (or any other tumour) was known at the time of testing [50, 87, 89], although the follow-up period was possibly too short in some to rule out a malignancy with certainty [50]. Graus et al. [36] did not find anti-Tr antibodies in 159 patients with cerebellar disorders without HD. Similarly, Probst et al. [89] found no evidence of anti-Tr/DNER in 25 patients with non-Hodgkin’s lymphoma—7 with breast cancer, 6 with non-SCLC, 3 with ovarian cancer and 13 with other tumours—in neurological and non-neurological patients without tumour or in healthy controls. anti-Tr is also absent in patients with PCD and non-Hodgkin’s lymphoma in another study [87]. Rarely, anti-Tr may be present in patients with HD but no neurological symptoms [36, 87].

Histological HD subtypes found in anti-Tr-positive patients included mostly nodular sclerosing HD but also cases with mixed cellularity or, least commonly, lymphocyte depletion [36, 50, 87]. HD stages at the time of first diagnoses ranged from I to IVA [36]. Limited HD (stage I or II) was the most common finding (76 %) in one of the largest series on anti-Tr-positive patients [50].

The diagnosis of PCD precedes the diagnosis of HD in the vast majority of patients [36, 50, 87]. The median time between PCD and HD diagnosis was 5 months (range, 0–12), 3.5 months (range 0–24) and 4.2 months in three independent studies [36, 50, 87]. However, PCD may rarely also develop after tumour diagnosis (median 12 months; range 3–120); in such patients, anti-Tr-positive PCD may predict HD recurrence [50].

Of special note, anti-Tr reportedly disappeared spontaneously in some patients without HD. Whether this was caused by immune-mediated eradication of the tumour (as sometimes observed in anti-Hu syndrome and SCLC) or whether anti-Tr is of non-paraneoplastic (e.g. post-infectious) origin in some cases remains to be elucidated.

#### Outcome and prognosis

In the index patient, radiation therapy for HD halted the progression of the neurological disease. In the study by Graus et al. [36] treatment with chemotherapy (in four) or radiotherapy (in one) resulted in complete remission of HD in all patients (median follow-up, 46 months; range, 12–52) and stabilization or remission of the neurological symptoms. However, cerebellar atrophy was present in all cases at last follow-up, and 4/5 patients were disabled due to the cerebellar syndrome. One patient showed complete remission within 2 months under treatment with corticosteroids. In the cohort of Bernal et al. [50], the cerebellar syndrome was subacute and irreversible in 22/26 patients (includes all three patients with anti-Tr but not tumour) with poor functional outcome but showed a clear improvement in the remaining four, suggesting that the cerebellar damage is reversible in some instances. One patient in that cohort stabilized following haematopoietic stem cell autotransplant. Of note, complete remission of the ataxia was found in 43 % of the patients under 40 years of age, whereas only 5 % older than 40 years showed at least partial remission [50].

In the study by de Graaf et al. [87], most patients showed little (4/11) or no (6) improvement on the modified Rankin score after tumour therapy and, in five, immunotherapy (PEX and/or IVIG); one patient worsened under treatment [87]. Complete tumour remission was achieved in 10/11 cases and stable disease in one.

#### Antigen

**Fig. 5:**
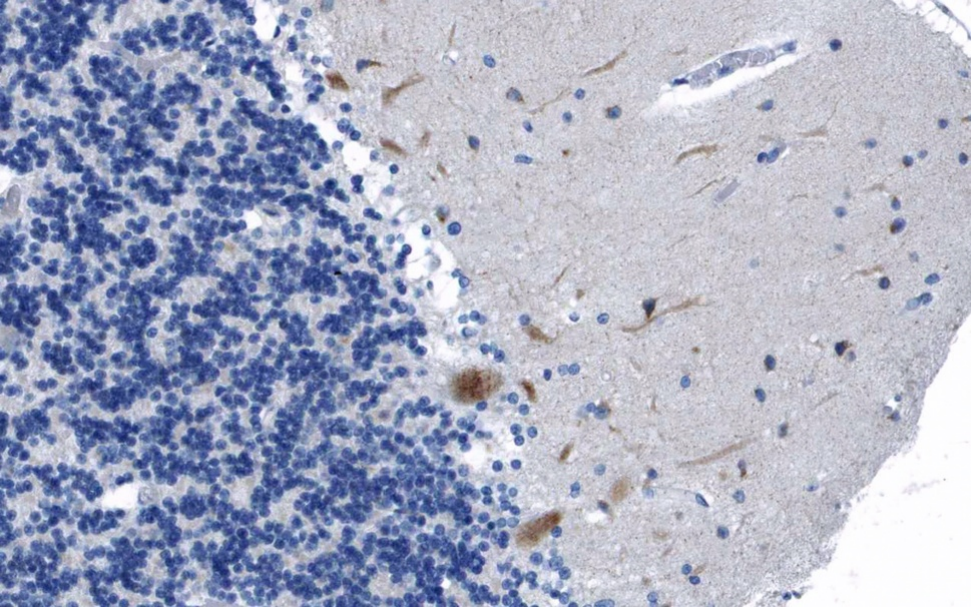
Expression of DNER in the human cerebellum as demonstrated by IHC (modified image from the *Human Protein Atlas* image database [33])

DNER, the target antigen of anti-Tr [87], is a single-pass type I transmembrane protein highly expressed in PC cell bodies and dendrites (but not axons) (Fig. 5) as well as in numerous other neurons, including cortical and hippocampal pyramidal neurons in mice [91]. Extracellular EGF repeats make it accessible to circulating anti-Tr. The main antigenic epitope has been indeed mapped to an extracellular 176-aa region between EGF domains 2 and 3. Some sera recognized a second epitope, which was also located in the extracellular region of DNER, but no differences in the clinical phenotype were noted [87]. DNER has also been detected intracellularly in the sorting endosomal compartment of dendrites and cell bodies in various types of CNS neurons [91]. As a Notch ligand activating the NOTCH1 pathway, it is thought to mediate PC-Bergmann glial interaction during cerebellar development [91–93]. Dner-knockout mice show cerebellar hypoplasia, irregular CF and PF innervation of PCs and lowered glutamate aspartate transporter (GLAST) expression in Bergmann glia, resulting in reduced glutamate clearance at the PC-PF synapses. Functionally, lack of Dner is associated with disturbed motor coordination in the fixed bar and rotarod tests [93]. Moreover, cerebellar histogenesis is retarded in DNER-knockout mice, resulting in overall size reduction, hypoplasia of the cerebellar fissure and folia, delayed layer organization with significantly more remaining granule cells in the extragranular layer and a thinner molecular layer than in wild-type controls [93].

#### Immunohistochemistry

**Fig. 6:**
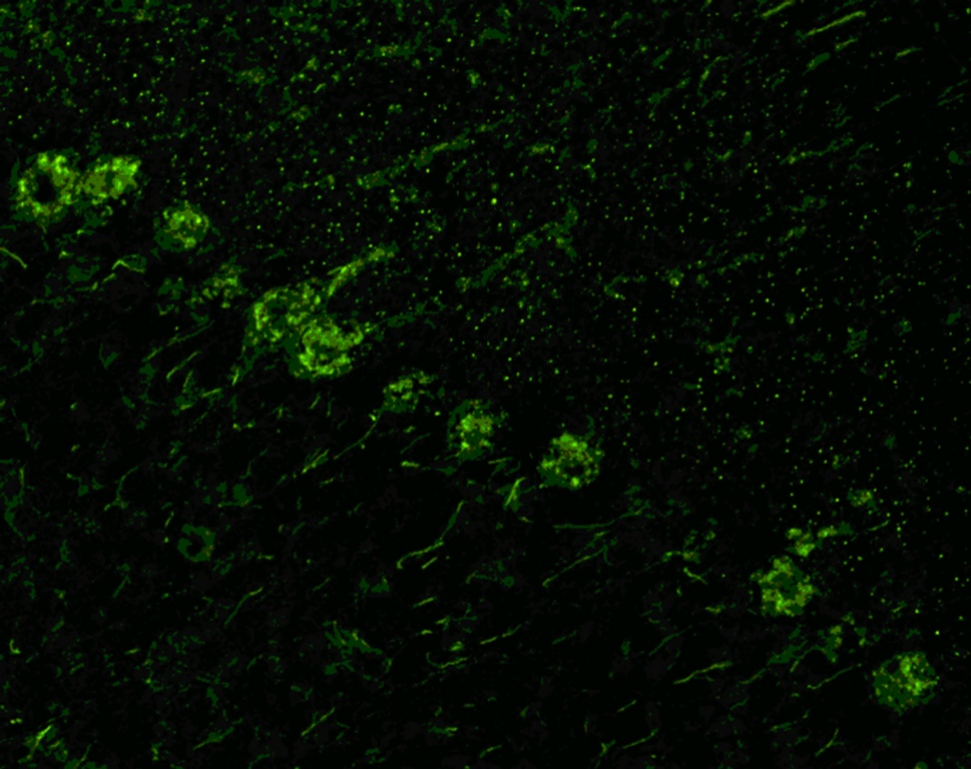
Binding of IgG from a patient with anti-Tr/DNER-associated ACA to a rat cerebellum tissue section. A goat anti-human IgG secondary antibody labelled with fluorescein isothiocyanate (*green fluorescence*) was used to visualize bound patient IgG

Anti-Tr were first identified by means of an indirect immunofluorescence assay employing unfixed human cerebellum tissue sections [88]. Graus et al. [36], in a study aiming to immunologically characterize the anti-Tr reactivity in more detail, used conventional IHC on acetone-fixed human and rat cerebellum sections. Both studies found labelling of the PC cell bodies and proximal dendrites [36, 88]. However, anti-Tr are best characterized by the fine dotted pattern they cause in the molecular layer, which probably corresponds to staining of small dendritic branchlets perpendicular to the plane of the section (Fig. 6) [36, 94]. The PC axons are usually spared [94]. The rat PC cytoplasmic staining pattern was described as coarse granular [36, 94]. Neurons of the deep cerebellar nuclei are only faintly positive [36, 50], which is in contrast to the strong staining observed with anti-Yo-positive sera [94]. While one study found no binding to basket and stellate neurons [36], another one did [94]. Bernal et al. [50] also located the punctuate staining to dendritic branchlets in the molecular layer of the cerebellum. Neurons of the granular layer are negative, except possibly in a few Golgi cells [36, 50, 94].

Of note, important differences in the extra-cerebellar binding pattern of anti-Tr were found between rat and human tissue [36]. In rat tissue, anti-Tr was found to bind not only to PCs but also strongly (though not in the dotted patterns observed in the cerebellum) to neurons in the hilus of the dentate gyrus and the plexiform layers of the hippocampus and to randomly distributed neurons in the molecular layer and layers II and III of the neocortex (but not to large neocortical projection neurons), to neurons in the piriform and entorhinal cortex, the lateral and basal amygdala, the trigeminal ganglia, the striatum, and less intensely, to neurons in the reticular thalamic nuclei of the thalamus, in the diencephalon and in the brainstem [36, 87]. By contrast, no anti-Tr immunoreactivity was observed in the large neocortical projection neurons, in glial and ependymal cells, in the choroid plexus and in the leptomeninges or in extra-neuronal tissues such as liver, kidney, spleen, thymus, adrenal cortex and testis.

In human tissue, anti-Tr only labelled PCs, in a pattern similar to that of anti-Yo, but not neurons in other CNS regions, including the cerebral cortex, hippocampus, putamen, thalamus, mammillary bodies, lateral geniculate body, substantia nigra and dentate nucleus [36].

The staining characteristic for anti-Tr is in line with the distribution of DNER, which is highly expressed in the PCs in a punctuate somatodendritic manner and has been shown to be also present in isolated pyramidal hippocampal neurons in rat brain sections [87, 91, 95].

Besides IHC, immunocytochemistry (ICC) employing primary hippocampal neurons has been used to demonstrate anti-Tr seropositivity: co-localization of patient IgG with a commercial anti-DNER antibody was taken as evidence for anti-Tr positivity [87, 96].

#### Antigen-specific assays

De Graaf et al. [87] successfully used a CBA employing formalin-fixed HeLa cells expressing full-length human influenza haemagglutinin-tagged DNER and non-transfected control cells to detect anti-Tr. This assay yielded a sensitivity of 100 % (CI 95 % 69.9–99.2; 12/12 samples) and a specificity of 99.59 % (95 % CI 97.4–100; 1/246 control samples). Of note, the only positive control serum was obtained from a patient with HD but no neurological symptoms and also showed anti-Tr positivity when tested by IHC. An independent study used both DNER-transfected HEK293 cells and HeLa cells as antigenic substrates to detect anti-Tr/DNER in 6 patients with HD. In that study, the use of living instead of fixed, permeabilized, transfected cells resulted in an improved signal-to-noise ratio [96]. Very recently, a commercial CBA using formalin-fixed HEK293 cells transfected with full-length human DNER and mock-transfected as well as CDR2/Yo and CDR2L-transfected cells as control substrates has been described, which yielded a sensitivity of 100 % (95 % CI 92.8–100 %) based on 38 anti-Tr-positive samples and a specificity of 100 % (95 % CI 98.7–100 %) based on 201 control samples [89].

Anti-Tr has also been detected by using wild-type hippocampal pyramidal neurons and neurons in which anti-Tr was knocked down with shRNA constructs [87]; accordingly, cerebellum sections from wild-type and DNER knock-out mice could be used as substrates for anti-Tr testing.

De Graaf et al. [87] mapped the main epitope to an N-glycosylated extracellular region of DNER. This was recently confirmed in an independent study reporting a lack of anti-Tr staining after preincubation of sera with the extracellular domain of DNER [89]. This is important for the development of future immunoassays for anti-Tr/DNER.

Of note, N-glycosylation mutations abolishes the anti-Tr staining, indicating that glycosylation of DNER is required for detecting anti-Tr [87, 95].

Knock-down of DNER by transfection with short hairpin RNA-producing constructs eliminated anti-Tr staining in hippocampal neuron culture, proving that DNER is the only antigen of anti-Tr. Moreover, preadsorption IHC experiments have been successfully applied. Incubation of anti-Tr sera with either biotinylated GFP-DNER coupled to streptavidin beads, recombinant full-length DNER or its extracellular domain prior to IHC resulted in a loss of anti-Tr-specific staining in two recent studies [87, 89].

Immunoblots of isolated PCs or mouse cerebellum homogenates have been attempted but failed to identify a common band both under reducing and non-reducing conditions [36, 97]. Similarly, Western blotting with either total extracts from HEK293 cells expressing HA-DNER or purified HA-DNER from HEK293 cells was unreliable for diagnosing anti-Tr [87]. Of 12 anti-Tr-positive sera tested, only 3 came up positive in the total extract-WB and only 11 in the purified DNER-WB.

Some studies used an epitope-blocking protocol to confirm that sera showing such binding patterns target the same antigen as previously identified sera [50]. Abolished binding of a biotinylated anti-Tr-positive immunoglobulin G (IgG) from an index patient by preincubation with undiluted serum from a patient with anti-Tr-like binding on rat cerebellum sections was taken as evidence for epitope identity [50].

#### CSF testing

Anti-Tr antibodies are mostly produced intrathecally as indicated by an elevated Tr-specific antibody index [36]. Of note, Bernal et al. [50] reported two rare cases (2/28; 7 %) in which the antibody was detected only in the CSF but not in the serum; however, a high screening serum dilution of 1:500 was used. As expected, CSF samples from anti-Tr-positive patients were shown to recognize DNER [87, 96].

#### Association with other autoantibodies

Antibodies to DNER were not found in 70 samples positive for either anti-Hu, anti-Yo, anti-Ri, anti-Ma, anti-amphiphysin or anti-CV2/CRMP5 [87]. Anti-DNER was also negative in 30 patients with systemic lupus erythematosus or rheumatic arthritis [87]. All anti-Tr sera tested by Graus et al. [36] were negative for anti-Hu, -Yo and -Ri antibodies, and none of the control sera with anti-Yo or other anti-Purkinje cell antibodies was positive for anti-Tr.

#### Pathogenetic relevance

The main epitopes of anti-Tr/DNER have been mapped to an N-glycosylated extracellular region of DNER by using deletion constructs and a glycosylation inhibitor [87]. Accordingly, binding of anti-Tr/DNER to non-permeabilized, living hippocampal neurons, which naturally express DNER on their surface, has been demonstrated [87, 96], rendering a direct pathogenic effect of anti-Tr/DNER at least possible. Alternatively, cerebellar damage in Tr/DNER might be caused by DNER-specific T cells.

The observation of severe PC loss in an anti-Tr-positive patient with ACA (but no tumour) in the absence of any significant cellular infiltrates suggests that anti-Tr potentially has a pathogenic impact [50]. Histopathology revealed only sporadic T cells and macrophages but a total loss of PCs accompanied by mild Bergmann gliosis. In addition, mild secondary neuronal cell loss with gliosis was noted in the dentate nucleus and inferior olive. In line with that finding, ataxia is irreversible or shows only little improvement in many anti-Tr-positive patients [87].

Further indirect findings in favour of a pathogenic role of anti-Tr include the strong association of anti-Tr with ataxia in patients with HD, with the antibody being virtually absent in patients with HD and no PCD, the intrathecal synthesis of anti-Tr, the observation that DNER knockout causes ataxia in mice, and from the predominance of complement-activating IgG1 and IgG3 antibodies among anti-Tr subclasses (which may suggest a Th1-type response of CD4^+^ T helper cells). All five patients reported by Graus et al. [36] were positive only for Tr-IgG (detectable also by anti-human kappa and lambda) but not for Tr-IgM. Bernal et al. [50] found anti-Tr-IgG1 (4/17) or -IgG3 (4) or both (9) in all tested sera. Two sera with anti-Tr-IgG2 or -IgG4 were also anti-Tr-IgG1 positive; again, no anti-Tr-IgM was detected [50].

On the other hand, no obvious abnormalities in morphology were observed in either cerebellar organotypic or primary hippocampal neuron cultures upon incubation with anti-Tr-positive sera [87]. However, it is unclear whether the cells were incubated together with human complement as required for antibody-dependent complement-mediated cytotoxicity, which has been shown to be the major pathogenetic mechanism in other antibody-mediated autoimmune disorders of the CNS such as NMO [98–100].

The relationship between HD and anti-Tr autoimmunity is not well understood. While most patients have HD, anti-Tr have also been described in some patients without tumour (follow-up time up to 3 years) [50]. Moreover, anti-Tr normally do not bind to pathological lymph nodes from patients with HD [36, 50] and, accordingly, DNER expression seems to be absent in HD tissue. A role of ectopic DNER expression as a disease trigger is thus unlikely.

Taking into consideration that HD may promote T-cell dysfunction, resulting in an increased risk for viral infection [36, 101], that HD is often associated with polyclonal B cell activation [102], and the observation that anti-Tr-related PCD was indeed preceded by febrile infections in some patients, a possible parainfectious origin of anti-Tr in the context of HD-related immune dysregulation has been proposed as an alternative explanation [50, 87].

Of note, successful treatment of HD is accompanied by disappearance of anti-Tr [50]. In addition, spontaneous disappearance in patients without tumour has been observed [50], which is different from what has been reported in patients with anti-Hu and anti-Yo antibodies [103–105].

#### Molecular genetics

So far, no association of SCA with mutations in the DNER gene has been reported in humans.

## Medusa head antibodies of unknown specificity

Over the past 4 years, we have seen a number of samples that showed a Medusa head pattern when tested by IHC but were neither positive for anti-mGluR1, anti-Homer-3, anti-ITPR1, anti-CARP VIII, anti-Ca/ARHGAP26, anti-GluRδ2, anti-Yo and anti-Tr/DNER when tested using commercial CBAs (Euroimmun) nor displayed the additional IHC and WB features reported for PCA-2 or anti-PKCγ. As some of those samples yielded high titres, it is unlikely that sensitivity issues underlie this observation in all cases. This suggests that antibodies to further somatodendritic PC antigens may exist.

## Other autoantibodies associated with ACA

Several other anti-neuronal and anti-glial autoantibodies have been reported in association with cerebellar ataxia. However, the immunostaining observed with those antibodies differs significantly from that described above in that they do not bind in a somatodendritic pattern.

While some of those antibodies bind to neuronal nuclei (anti-Hu, anti-Ri, anti-ANNA3) or nucleoli (anti-Ma2/Ta), most bind to cytoplasmic or plasma membrane-bound antigens. Staining of intestinal tissue sections can be helpful when it comes to discriminating these antibodies. While Hu antibodies also bind to the cell nuclei of the plexus myentericus neurons, Ri antibodies do not.

Anti-GAD antibodies are characterized by strong binding to the granular layer. Anti-amphiphysin, anti-DPPX and anti-GABABR antibodies show a similar pattern to that of anti-GAD antibodies, but in addition react with the neuropil of the molecular layer while sparing the PC somata. While anti-amphiphysin tend to stain more strongly the molecular layer than the granular layer, the opposite is true for anti-DPPX antibodies [106–108]. The binding pattern of anti-GABABR is less well defined since sera may react either with pre-synaptic receptors located on PF or with post-synaptic receptors present on PC fibres, or both. In addition, the two anti-voltage-gated potassium channel (VGKC)-complex antibodies CASPR2 [109, 110] and LGI1 [111] have both recently been implicated in ACA.

Anti-glial cell reactivities include antibodies to AQP4, the most important laboratory marker of NMO [112, 113], and so-called anti-Bergmann glial cell nuclear antibodies (AGNA), which are considered markers of lung cancer. Manifestations of AQP4-Ab-positive NMO only rarely include cerebellar symptoms, some of which are caused by lesions in the cerebellar pedunculi. AQP4-IgG bind in a very characteristic pattern to astrocytic endfeet adjacent to the blood vessels, Virchow Robin spaces and pia mater. In addition, chicken-wire-like staining of the granular layer can be present, probably corresponding to the astrocytic sheath of cerebellar glomeruli. Anti-SOX1 is a marker of SCLC and was originally thought to be identical to AGNA antibodies, while recent observations rather suggest a co-reactivity of two independent antibodies.

Anti-CV2/CRMP5 is a common reactivity in patients with paraneoplastic neurological disorders and typically produces a ‘sand-like’ staining pattern, most prominently in the cerebellar white matter.

Antibodies to transglutaminase 6, which some authors consider a frequent cause of cerebellar ataxia that possibly can be treated by gluten-free diet [114, 115], are not normally diagnosed by IHC.

Furthermore, a number of (so far mostly unconfirmed) post-infectious antibodies have been described in the literature, including anti-centrosome antibodies (anti-γγ-enolase, anti-pericentrin, anti-ninein, anti-PCM1 and anti-Mob1) in patients with post-varicella ACA [116], anti-centriolar antibodies in post-*Mycoplasma pneumoniae* ACA [117] and antibodies to triophosphate in patients with post-Epstein-Barr virus ACA [118].

Finally, an anti-GD1b IgG-associated, atactic Guillain-Barré syndrome subvariant has been proposed [119–121]; however, its nosological status as a cerebellar disorder is controversial [122].

## Diagnostic pitfalls and limitations

While IHC is an important screening method when it comes to testing patients for anti-neural antibodies, its specificity is limited.

First, the IHC staining patterns described for the 12 ‘Medusa head’ antibodies discussed above are rather similar. Accordingly, it is difficult for the non-expert rater to correctly distinguish the various patterns, especially at the lower magnification often employed in routine diagnostic laboratories. The use of additional tissues may facilitate differentiation of the various antibodies.

Second, co-existing autoantibodies present in the patients’ serum or CSF may alter the typical binding patterns described above. Anti-neural antibodies may either occur in association with other anti-neural antibodies or with non-CNS-specific, pathogenic or non-pathogenic (e.g. naturally occurring) systemic autoantibodies. This can produce atypical staining patterns and may delay making the correct diagnosis. For example, ANA, which are frequently present even in healthy subjects, can cause staining of the interneurons of the molecular layer, a feature not present with anti-Ca/ARHGAP26, anti-Sj/ITPR1 and other known PC antibodies, resulting in atypical IHC patterns. This underlines the importance of additional antigen-specific testing. In some cases, use of CSF instead of serum or titration of serum or CSF samples may reduce confusing co-staining by co-existing antibodies. However, titration may also result in the loss of diagnostically important details if antigen concentrations vary between cells or subcellular structures. For example, axons are less strongly stained by anti-ARHGAP26 than PC dendrites and might not be visible if sera are diluted. Alternatively interference by non-CNS-specific antibodies can be reduced or eliminated by pre-adsorption with acetone liver powder [112, 123, 124], a rather crude procedure that may also result in a loss in sensitivity.

Third, binding patterns on cerebellar tissue sections may vary between species. For example, anti-Tr antibodies have been found to bind to both human and rat cerebellum but exclusively to rat hippocampus [36, 125]. Similarly, many other rat brain tissues stained by anti-Tr are not reactive if human tissue is used. Differences between species have also been observed with anti-Yo [34, 39, 126].

**Fig. 7:**
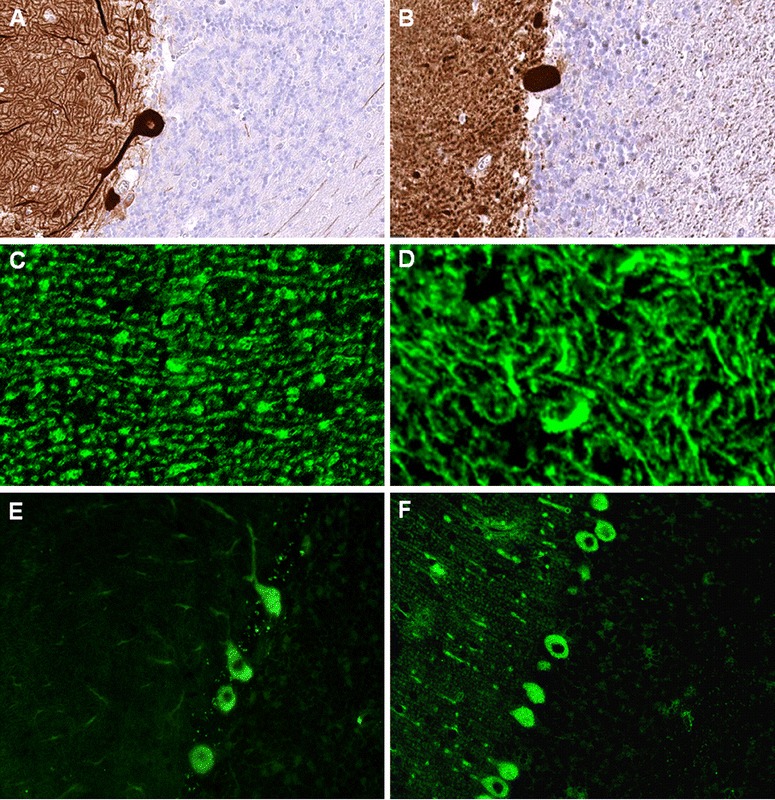
IHC patterns strongly depend on sectional planes and tissue donor species. **a**, **b** Binding of a commercial Homer-3 antibody to different sectional planes of human cerebellum tissue (modified images from [33]). **c**, **d** Binding of human IgG from a patient with ITPR1-Ab-positive ACA to mouse (**c**) and monkey (**d**) PC dendrites, respectively, sectioned at different planes. **e**, **f** Anti-Yo IgG antibodies as detected by use of primate (**e**) and mouse (**f**) cerebellum tissue sections, respectively, sectioned at different planes

Fourth, tissue morphology can differ substantially depending on sectional planes, which can be confusing for raters not familiar with the complex cerebellar cytoarchitecture (Fig. 7).

Fifth, IHC binding patterns may depend on techniques used for tissue preservation/fixation, which can critically alter target antigen confirmation, and antigen retrieval. For example, anti-CARP VIII were only detected on frozen tissue sections but not on paraffin-embedded sections [127]. Using thin tissue sections, intracellular antigens can usually be detected without a need for cell permeabilization since most PCs will be transected. Similarly, some CBAs strongly depend on fixation.

Sixth, expression levels of some antigens differ between PC somata, dendrites and axons. In consequence, structures which contain only low levels of the respective antigen, in particular the PC axons, might not get stained by low titre samples or when samples are tested at high dilution (e.g. to reduce background staining).

Given those limitations, IHC testing should ideally be followed by antigen-specific testing, even if results are *prima facie* considered typical. In addition to immunoblot assays, CBAs are increasingly being used to detect anti-neuronal antibodies. While solid data on the sensitivity of IHC testing for most of the anti-PC antibodies discussed here are lacking, recombinant assays have been shown to be more sensitive than IHC in some other autoantibody-associated diseases of the CNS [128]. A very recent study found a higher sensitivity of an anti-Tr/DNER-specific CBA in a direct comparison with IHC [89].

Competitive IHC based on abolition of patient IgG binding by preincubating the respective diagnostic substrate with a strongly binding positive reference serum [50] as well as pre-adsorption of patient sera with recombinant antigen have both been successfully used to confirm antigen specificity [129, 130]. IHC is not routinely used for detecting anti-VGCC, which are usually tested for by RIA with native protein as antigenic substrate.

As very low-titre antibodies might not be detectable by IHC, it may be recommendable to use antigen-specific assays in IHC-negative cases, too. However, it must be kept in mind that the diagnostic specificity of some of the currently available antigen-specific immunoassays, many of which are in-house assays, has not been evaluated in sufficiently large cohorts and false-positive results cannot be excluded.

In the case of discrepant results between IHC and antigen-specific testing, a third, methodologically independent assay should be used. In addition (or alternatively, if no third method is available), follow-up testing should be considered, though this may not rule out false-positive results caused by systematic confounders related to the respective detection method.

Titres may decline after PEX, immunosuppressive or steroid treatment, or chemotherapy for cancer and may temporarily drop below detection limits. Re-testing later in the disease course is therefore recommended in ‘seronegative’ cases if initial testing was done during periods of active treatment.

As discussed above, more autoantibodies to PCs may exist than known to date. Accordingly, negative test results obtained with recombinant assays in patients with ‘Medusa head ataxia’ do not necessarily indicate insufficient sensitivity of the respective assay; instead, the antibodies might target a so-far unknown antigen.

Many immunoassays, including IHC and CBAs, are rarely affected by the so-called high-dose hook effect (HDE), which results in false-negative or falsely low results when low dilutions are used. While that effect is thought to be caused by a saturating excess in antigen concentration in ELISA preventing sandwich or bridge formation, the cause of HDE in IHC and ICC is less well understood. It has been speculated that HDE in IIF might be caused by anti-immunoglobulin conjugates being unable to reach their antigenic determinants on tightly clustered immunoglobulin molecules [112, 131]. Samples should therefore be tested at two or more different dilutions.

Finally, it should be kept in mind that some of the PC antibodies reported so far were identified by screening of pre-selected cohorts of patients with ACA. Moreover, some of the identified target antigens are expressed outside the cerebellum as well. Accordingly, the full spectrum of clinical signs and symptoms associated with those antibodies may expand in the future when more patients become tested. Similarly, a broader spectrum of symptoms than initially thought has been found in many other antibody-associated diseases of the CNS over the past few years [106, 110, 113, 132–135]. In consequence, the presence of signs or symptoms other than cerebellar ataxia does not per se argue against the validity of a given test result.

That said, it is important to consider that the ratio of false-positive to true-positive results found with any test depends on the number of subjects tested that do not have the respective disease. In order to limit the risk of false-positive results, we therefore do not currently recommend testing routinely for any of the antibodies reviewed here using recombinant assays in indications other than those outlined above, unless qualitative evidence from IHC assays clearly indicates the presence of antibodies to PCs.

## Differential diagnoses

While autoantibody-associated autoimmunity is an important differential diagnosis in patients presenting with signs or symptoms of cerebellar ataxia, other causes need to be considered as well. These include the many forms of hereditary ataxia as well as toxic (including chemotherapy-related), infectious and vascular diseases of the cerebellum. In addition, other forms of ACA have to be taken into account, including, among others, multiple sclerosis, MOG-IgG-related autoimmunity, (very rarely) neuromyelitis optica, rheumatic disorders and vasculitis, Behçet disease, ADEM and post-infectious cerebellitis with presumed T cell-mediated pathogenesis, and sarcoidosis. An overview of autoantibodies to neuronal or glial cells other than PCs that have been reported in patients with ACA is given in Table I in the first part of this article series.

## Summary and outlook

The past few years have seen the discovery of a multitude of new autoantibodies and autoantigens in patients with cerebellar ataxia. Many patients previously diagnosed with ‘idiopathic ataxia’ can now be given a more definite diagnosis. Of note, some of these novel reactivities not only indicate an autoimmune pathogenesis but were also shown to predict a facultative or obligate paraneoplastic aetiology.

While in some disorders, such as anti-mGluR1 and anti-VGCC ACA and, possibly, anti-GluRδ2 ACA, evidence for a directly pathogenic role of the antibody exists, it is unknown in others whether the antibody itself is harmful or only an epiphenomenon of diagnostic rather than pathogenic relevance. Alternatively, a T cell-mediated immune response may cause damage to the cerebellum in those disorders.

It has been argued that antibodies to intracellular antigens (as opposed to antigens expressed on the cell surface) are likely not directly involved in the pathogenesis of ACA since they may not be capable of reaching their target antigens. However, passive transfer experiments, which could only prove that hypothesis, have not yet been performed in many of those syndromes. Moreover, uptake of human [32, 54–59, 136–138] as well as mouse [139] IgG by PCs has been described in numerous studies. Given that most of the intracellular antigens targeted by those antibodies are linked to calcium homoeostasis as outlined in this article, which is crucial for many cell functions and dysregulation of which may result in cell death, including by apoptosis [140], a direct effect of antibodies to intracellular antigens on cell function and survival cannot be fully ruled out. Incorporation of anti-CDR2 and CDR2L antibodies (anti-Yo) has recently indeed been reported to affect PC calcium homoeostasis [32] and to cause defective PC arborization [32] and PC cell death in cultured PCs [57], though confirmatory studies are certainly needed.

Moreover, antibodies against intracellular antigens have been previously shown to be of pathogenic impact in syndromes other than ACA. Injection of human serum containing high titres of antibodies to amphiphysin, a protein associated with the cytoplasmic surface of synaptic vesicles, was found to cause dose-dependent stiffness, with spasms resembling human stiff-person syndrome in rats [141–143] as well as anxiety behaviour [144]. IgG to amphiphysin was shown to be internalized by neurons and to alter the function of inhibitory synapses in vivo [142]. Amphiphysin is also a known autoantigen in PCD. These findings might potentially be of relevance also for ARHGAP26-related ACA. ARHGAP26 and amphiphysin are both involved in endocytosis, and anti-ARHGAP26 has been shown to co-precipitate dynamin, an important binding partner of amphiphysin, from mouse and rat cerebellum or brain extract [130, 145]. Similarly, antibodies to recoverin have been demonstrated to enter retinal cells, probably by endocytosis, where they induce caspase-dependent apoptosis [146]. A pathogenic effect has also been proposed for antibodies to the intracellular antigen glutamate decarboxylase 65 (GAD65) [147, 148]. Finally, endocytotic uptake of antibodies has also been found for cell types other than PCs and for a subset of anti-DNA antibodies [149–152].

Interestingly, PCs have been reported to incorporate IgG and IgM irrespective of the immunoglobulin’s reactivity with Purkinje cell surface antigens [56], abolishing the need for surface expression as a prerequisite of antibody uptake.

The fact that so many novel autoantigens have been detected over the past few years and the finding of PC-specific staining in a substantial number of patients with ACA negative for all currently known antibodies renders it likely that the future will see the identification of even more target proteins in this condition. This offers great diagnostic opportunities but poses also many new diagnostic challenges: While IHC was previously the mainstay when it came to diagnosing anti-neuronal antibodies, when anti-Yo and anti-Tr were the only clinically relevant somatodendritic PC reactivities, the increasing number of known autoantibodies staining PC somata and dendrites has made the use of recombinant assays crucial. Each of these assays has its specific advantages and disadvantages and needs to be carefully evaluated—also in comparison with other assays. Given the broad spectrum of methods that can be used for detecting autoantibodies, developing recombinant immunoassays that are both sufficiently sensitive and specific and feasible for routine clinical use can be an ambitious endeavour as exemplified by the changeful and troublesome history of MOG-IgG testing in multiple sclerosis and related disorders. This is all the more true in conditions of (obligately or facultatively) paraneoplastic origin, in which false-negative results may cause a significant delay in tumour diagnosis and treatment and in which false-positive results may submit patients to unnecessary and potentially harmful diagnostic procedures. Given the multitude of in-house assays currently used for diagnosing ACA-associated autoantibodies, the development of standardized assays as well as systematic inter-laboratory comparisons are highly warranted.

In summary, autoantibody-associated ACA is a disorder that was probably underdiagnosed in the past. The discovery of PC-specific autoantibodies has shed new light on the pathogenesis of ACA, and the development of recombinant assays has facilitated the early and specific diagnosis, and thus therapy, of this rare but often devastating condition.

## Abbreviations

Aa: amino acids
ACA: autoimmune cerebellar ataxia
AChR: acetylcholine receptor
ADEM: acute disseminated encephalomyelitis
AF: Alexa Fluor
AGNA: anti-glial cell nuclear antibodies
AMPA: α-amino-3-hydroxy-5-methyl-4-isoxazolepropionic acid
ANA: anti-nuclear antibodies
ANNA: anti-neuronal nuclear antibody
APTX: aprataxin
AQP4: aquaporin-4
ARHGAP26: rho GTPase-activating protein 26
ATXN1: ataxin-1
BAR: bin–amphiphysin–Rvs
beta-NAP: neuronal adaptin-like protein
CARP VIII: carbonic anhydrase-related protein VIII
CASPR2: contactin-associated protein-2
CBA: cell-based assay
cDNA: complementary DNA
CDR2: cerebellar degeneration-related protein 2
CDR2L: CDR2-like
CDR3: cerebellar degeneration-related autoantigen-3
CF: climbing fibres
CHO: Chinese hamster ovary
CNS: central nervous system
CSF: cerebrospinal fluid
CTL: cytotoxic T lymphocytes
DAG: diacylglycerin
DAPI: 4′,6-diamidino-2-phenylindole
DNA: deoxyribonucleic acid
DNER: delta notch-like epidermal growth factor-related receptor
DPPX: dipeptidyl-peptidase 6
EA2: episodic ataxia type 2
EGF: epidermal growth factor
ELISA: enzyme-linked immunosorbent assay
ER: endoplasmic reticulum
FHM1: familial hemiplegic migraine 1
FITC: fluorescein isothiocyanate
GABA: γ-aminobutyric acid
GABABR: GABA type B receptor
GAD: glutamic acid decarboxylase
GAP: rho GTPase-activating protein
GFAP: glial fibrillary acidic protein
GL: granular layer
GluR: ionotropic glutamate receptors
GluRδ2: glutamate receptor delta 2
GRAF1: GTPase regulator associated with focal adhesion kinase 1
HEK: human embryonic kidney
IB: immunoblot assay
ICC: immunocytochemistry
IIF: indirect immunofluorescence
IgA: immunoglobulin A
IgG: immunoglobulin G
IgM: immunoglobulin M
IHC: immunohistochemistry
IP: immunoprecipitation assay
IP3: inositol 1,4,5-trisphosphate
IRBIT: IP_3_ receptor-binding protein released with IP3
ITPR1: inositol 1,4,5-trisphosphate receptor, type 1
IVIG: intravenous immunoglobulins
IVMP: intravenous methylprednisolone
kDa: kilodalton
LEMS: Lambert–Eaton syndrome
LGI1: leucine-rich, glioma inactivated 1
LTD: long-term depression
LTP: long-term potentiation
mGluR1: metabotropic glutamate receptor 1
ML: molecular layer
MRI: magnetic resonance imaging
mRNA: messenger RNA
NMDA: N-methyl D-aspartate
NMO: neuromyelitis optica
NSCLC: non-small cell lung cancer
NSE: neuron-specific enolase
OCB: oligoclonal bands
PC: Purkinje cell
PCA: Purkinje cell antibody
PCD: paraneoplastic cerebellar degeneration
PCL: PC layer
PF: parallel fibres
PIP_2_: phosphatidyl 4,5-bisphosphate
PKCγ: protein kinase C gamma
PLCβ3: phospholipase Cβ3
PSD: postsynaptic density
RACK: receptors for activated C kinases
RhoA: ras homolog gene family, member A
RIA: radioimmunoprecipitation assay
RNA: ribonucleic acid
SCA: spinocerebellar ataxia
SCLC: small-cell lung cancer
SOX: sex-determining region Y-box
TRPC: canonical transient receptor potential channel
TUNEL: TdT-mediated dUTP-biotin nick end labeling
VGCC: voltage-gated potassium channel
VGKC: voltage-gated potassium channel
WB: Western blot
WM: white matter
